# Pet serine protease from enteroaggregative *Escherichia coli* stimulates the inflammatory response activating human macrophages

**DOI:** 10.1186/s12866-016-0775-7

**Published:** 2016-07-20

**Authors:** L. M. Rocha-Ramírez, U. Hernández-Chiñas, D. Baños-Rojas, J. Xicohtencatl-Cortés, M. E. Chávez-Berrocal, G. Rico-Rosillo, R. Kretschmer, C. A. Eslava

**Affiliations:** Departamento de Infectología, Hospital Infantil de México Federico Gómez, Dr. Márquez No. 162, Col Doctores, Delegación Cuauhtémoc, 06720 C. de México; Departamento de Salud Pública, Facultad de Medicina, Universidad Nacional Autónoma de México, Circuito Escolar S/N, Ciudad Universitaria, Coyoacán, 04510 C. de México; Laboratorio de Patogenicidad Bacteriana, Unidad de Hemato-Oncología e Investigación, Hospital Infantil de México Federico Gómez/Facultad de Medicina UNAM, Dr. Márquez No. 162, Col Doctores, Delegación Cuauhtémoc, 06720 C. de México; Laboratorio de Bacteriología Intestinal, Unidad de Hemato-Oncología e Investigación, Hospital Infantil de México Federico Gómez, Dr. Márquez No. 162, Col Doctores, Delegación Cuauhtémoc, 06720 C. de México; Divisiòn de Investigación. Facultad de Medicina, UNAM. Circuito Escolar S/N, Ciudad Universitaria, Coyoacán, 04510 C. de México; Unidad de Investigación Médica en Inmunología, Hospital de Pediatría, CMN siglo XXI, IMSS, Av. Cuauhtémoc No. 330, Col Doctores, Delegación Cuauhtémoc, 06720 C. de México

**Keywords:** Serin protease, *Escherichia coli*, Innate immune response, Cytokines

## Abstract

**Background:**

Pet is a toxin from the family of Serine Protease Autotransporters of *Enterobacteriaceae* which was initially identified in Enteroaggregative *Escherichia coli* strains. This protease exhibits enterotoxin properties, damages the cell cytoskeleton and induces intestinal epithelium alterations, which are associated with a severe inflammatory process. An in-vitro study was conducted to evaluate the effect of Pet on the migration of human peripheral blood monocytes-derived macrophages and its participation in the activation of the early inflammatory response and cytokine expression.

**Results:**

In the macrophage migration activation assay, Pet produced a similar effect to that induced by opsonized zymosan (ZAS). Regarding the cytokine expression, an increase of IL-8, TNF-α (pro-inflammatory) and IL-10 (anti-inflammatory) was identified. In addition to the above results, the nuclear translocation of NF-kB pp65 was also identified. These events are probably related to the inflammatory response identified in the histological examination of intestine rat samples inoculated with Pet during a ligated loop assay.

**Conclusion:**

The results showed that Pet participates as an immunostimulant molecule for macrophages, which activates both their mobility and cytokine expression. These observations suggest that the toxin participates in the inflammatory process that is observed during the host infection by EAEC Pet producing.

## Background

Enteroaggregative *Escherichia coli* (EAEC) is a pathogen responsible of different cases of diarrhea, which include acute, persistent, bloody, traveler’s diarrhea and also diarrhea in HIV/AIDS patients [1]. Clinical and laboratory data suggest that the EAEC infection is associated with the inflammation of intestinal mucosa [2]. In a study on EAEC pathogenic mechanisms using an animal model (gnotobiotic pigs) inoculated with different strains isolated from infected patients, in different histological sections were observed intestinal damage characterized by shortening and rounding of intestinal microvilli from enterocytes without apparent mononuclear cell infiltration in the mucosa [3]. Others researches observed in a murine model that EAEC 042 (O44:H18) strain infection induced hypersecretion of mucus, mild inflammatory response with mononuclear infiltration and edema in the lamina propria of the intestine [4]. Epidemiological studies on EAEC infection in humans, showed the presence of lactoferrin, IL-8 and IL-1β in feces of the infected people [5–7]. In order to identify which components of the bacterium could be involved in the activation of the cytokine expression, in different in-vitro assays using HT29 or T84 intestinal cell lines infected with the EAEC O42 strain, was observed that the flagellin [8], the aggregative adherence fimbriae (AAF) [9], as well as basolateral disruption of epithelial cells [10] promoted the IL-8 release.

Pet (Plasmid encoded toxin) is a protease which belongs to the Serine Protease Autotransporters of *Enterobacteriaceae* (SPATEs) initially described in the EAEC O42 strain [11]. The in-vitro assays that have been performed to understand the biological properties of Pet show that it is a protease which induces cytotoxic and cytopathic effects on different cultured cell lines. When the cell damage was analyzed, it was identified that the alteration of the cytoskeleton structure was an important factor in the detachment of cells from the glass substratum [12]. In a cell fractionation assay it was identified that the alterations induced by Pet on the cytoskeleton were related to its capacity to degrade the fodrin protein [13]. Studies of patients with inflammatory bowel disease (IBD), Crohn’s disease and cancer [14], the isolation of different SPATEs-producing-*E.coli* strains was observed in more than a half of the samples. On the other hand, in the histological examination of samples from patients with Crohn’s disease, the accumulation of inflammatory cells, mainly macrophages M1, has been observed, which determines the migration of cell subpopulations, mainly TH1, as well as the expression of inflammatory mediators such as nitric oxide [15, 16].

The macrophages are a heterogeneous cell population with a great plasticity that are able to stimulate primary immune response through their PRRs groups such as the TLRs, lectins and other receptors from these cells [17]. The PAMPs-PRRs interaction activates the macrophages, which increases their mobility and induces the expression of costimulatory molecules as CD40 and ICAM that contributes to the production of specific cytokines. All these events allow an adequate immune response in the host [18].

Even if it was observed that the Pet activity participates in the chronic inflammatory response during the EAEC infection, the effect of the toxin in the innate immune response has not been known so far. In order to explore the Pet role in the innate immune response, it was analyzed if the toxin contributes to an increase of the migration and activation of human macrophages through the synthesis of early and late response proteins such as IL-8, TNF, IL-10 and the translocation of pp65 to NF-kB.

## Methods

### Bacterial strains

EAEC O42 (O44:H18) strain isolated from a child with diarrhea in Lima, Peru [19]. Minimum clone of Pet (pCFN1) generated by cloning *pet* from EAEC O42 strain into *E. coli* HB101 [11]. The EAEC O42 strain was grown in agar and Luria broth (LB) without antibiotics; the minimum clone was grown and maintained in the same medium plus ampicillin (100 μg/mL).

### Preparation and purification of Pet

Pet was obtained from the minimum clone (*E. coli* HB101 pCFN1) according to the method previously described [13]. Briefly, the strain HB101 (pCEFN1) was inoculated in tubes with 3 ml of LB plus ampicillin (Amp) and incubated for 6 h at 37 °C with constant stirring (Newbrownskii) at 200 rpm. Then, 1 ml of the first culture was added into 1 L of fresh LB + Amp medium and the incubation was continued for 18 h at 37 °C with constant stirring (200 rpm). The culture was centrifuged (Sorvall RC5) at 10,000 g at 4 °C for 30 min. The supernatant was precipitated with ammonium sulfate at 60 % of saturation (361 g of salt/L) and was kept at rest overnight at 4 °C. The precipitated proteins were concentrated by centrifugation (Sorvall RC5) at 10,000 g at 4 °C for 30 min. The protein pellet was suspended in sodium phosphate buffer (0.07 M, pH = 8.2) and dialyzed during 2 days with buffer changes every 12 h. Afterwards, proteins were dialyzed in 0.05 M Tris-HCl plus 0.01 M EDTA (pH = 8.0). The protein precipitate was subjected to an anion exchange chromatography (Q-sepharose. Pharmacia, USA), using buffer A (0.05 M Tris-HCl plus 0.01 M EDTA, pH = 8.0) and buffer B (0.5 M NaCl). The first peak of the Q-Sepharose chromatography was subjected to another separation by using the cation exchanger resin Mono S HR 5/5 (Pharmacia, USA) under moderate pressure and rapid flow (FPLC) using the buffers A and B. The elution profile was analyzed at 280 nm. The protein concentration was quantified using a microplate by the Bradford method; bovine serum albumin was used as standard. Colorful reaction was measured in a spectrophotometer [20] at 595 nm (Bio-Rad Protein Assay, cat number 500–0001). The purified protein was stored at−20 °C until its use.

### LPS elimination (endotoxin) by polymyxin-B agarose gel

The contained endotoxin in Pet preparations was eliminated using polymyxin-B agarose gel (Detoxi Gel, Pierce), according to the recommended instructions by the supplier. Briefly, aliquots of 0.5 mL polymyxin-B in agarose were dropped on the polypre columns of 0.5 mL (Pierce). The column was equilibrated by washing; five volumes phosphate buffer solution (PBS) and ten volumes 1 % sodium deoxycholate. A 250 μL aliquot of Pet (300 μg/mL) was placed on the column and was kept at room temperature for 60 min. The column was eluted with PBS and from this were collected 250 μL of toxin (Pet). Protein concentration was determined by the Bradford method and its purity was analyzed by polyacrylamide gel electrophoresis (SDS-PAGE).

### Endotoxin activity

Protein free of endotoxin was evaluated by the Limulus Amebocyte Lysate (LAL) assay (Cat. N-289–06, LONZA), following the manufacturer's recommendations. Briefly, 100 μL of Pet protein fraction was mixed with 100 μL of LAL and incubated for 60 min at 37 °C; the assay was performed in duplicate. The endotoxin concentration was established by comparing the data obtained in the negative and positive endotoxin controls. Values of 0.125 EU/mL or less were considered suitable for the usage of Pet in assays of toxin activity.

### Ligated loop model in rats

Sprague Dawley Rats (*n* = 4) weighing between 100 and 150 g, both sexes, were used for a ligated loop model. The experimental animals were obtained from CICUAL (CINVESTAV-IPN Animal Ethics Committee) and the assays were carried out according to the Official Mexican Standard NOM-062-ZOO-1999 (Norma Oficial Mexicana), which specifies the lawful techniques for the production, care and use of laboratory animals. In preparation of intestinal loops, the animals first received the anesthetics Xylazine (6.5 mg/kg) and Ketamine (34.5 mg/kg). Once the rats fell asleep, laparotomy and exposure of the small intestine were performed. Three segments of intestine were prepared of approximately 10 cm of length with an intermediate of 3 cm between each loop. To form the loops, a sterile surgical suture (# 0) was used. Each segment was inoculated with 1000 μL of 1.5 × 10^8^ CFU of Pet producing-*E coli* O42 strain. In alternative assays, the segments of terminal ileum were inoculated with 100 μg of purified Pet; PBS was used as negative test control. After inoculation of intestine segments, they were taken back to the abdominal cavity, the incision was stitched with the same surgical suture and the animals were left in the presence of sterile water for its consumption *ad libitum*. Twelve hours after having done the described treatment, the animals (prior anesthesia with Xylazine 6.5 mg/kg) were sacrificed by cervical dislocation and the ileum segments were dissected. The intestinal content was removed and afterwards, segments of approximately 1.0 cm were obtained for histological analysis. The intestinal samples were fixed with buffered formalin (pH 7.3, 10 %) and were embedded in paraffin. Serial sections of approximately 5 μm thick were cut and fixed on the glass slides. The preparations were stained with hematoxylin and eosin according to the procedures used by the Biological Stain Commission.

### Culture and preparation of human peripheral blood-derived macrophages

Mononuclear cells were obtained from the leukocyte concentrates bags (Buffycoat) from samples of four donors (according to the approval guidelines by Bioethics Committee) from the blood bank of Hospital Infantil de México Federico Gómez. The mononuclear cells fraction was obtained by gradient centrifugation Lymphopred™ (Nycomed AS, Oslo, Norway). Once separated, it was washed three times with physiological saline solution and obtained monocytes were purified by the negative selection method using a mixture of antibodies anti-CD3, CD7, CD19, CD45RA, CD56 and anti-IgE (MiltenyiBiotec, BergischGladbach, Germany) according to the manufacturer’s indications. The purity of monocytes was evaluated by flow cytometric analysis using anti-CD14 conjugated to FITC (Pharmingen, San Diego, CA, USA). Purified monocytes (3 × 10^5^ monocytes/mL) were differentiated to macrophages through culturing the cells for 7 days in low attachment plates of 24 wells (Costar, Corning, NY, USA) in RPMI-1640 medium supplemented with 10 % of fetal bovine serum (FBS) (Gibco, Invitrogen USA).

### Chemotaxis

The Pet chemotactic effect was analyzed in Boyden chamber of one well with double filter (Neuroprobe Inc, USA). The chamber was prepared by placing one filter of nitrocellulose with a diameter of 0.5 μm (Nucleopore) in the upper compartment and a cellulose nitrate filter with 8 μm on the bottom which was used as a receptor of migrated cells. A suspension of approximately 8 × 10^5^ mononuclear cells in 0.5 mL of Hanks’ solution were placed in the upper chamber; in the lower chamber, 1.7 mL of Hanks’ solution with 5, 10 or 20 μg/mL of Pet were applied. Opsonized zymosan (ZAS) and Hanks’ solution with BSA (0.2 %) were used as the positive and negative controls, respectively. The chambers were incubated up to 90 min at 37 °C in atmosphere of 5 % CO_2_. After the chambers had been emptied, the filters were removed and the cells adhered to the bottom of the lower chamber filter were fixed with 80 % ethanol during 15 min at room temperature. Then, cells were stained with Harris hematoxylin and the membranes were hydrated first with ethanol-Xylene (1:1) and later with Xylene (100 %). The membranes were placed over the slides and fixed with balsam resin from Canada. The cell count was performed by three different readers in an optical microscope (400X) and the count was analyzed in 5 different fields. The chemotaxis was expressed as the percentage of migrating cells.

### Cytokine expression

The previously obtained macrophages were challenged with different Pet concentrations (5, 10 and 20 μg/ml) for 6 and 24 h. The concentration of pro-inflammatory cytokines (IL-8, TNF-α) and anti-inflammatory (IL-10) from the supernatant of each sample were measured by ELISA with capture and antibodies detection (BD Pharmingen, San Diego, CA, USA), according to the manufacturer’s indications. Briefly, the ELISA plates (Costar) were treated with 100 μL of capture antibody of each cytokine diluted (1:250) in carbonate buffer pH 9.2. They were incubated overnight at 4 °C, then, the antibody was eliminated and 100 μL of blocking solution (10 % FBS in PBS) was added to each well and further incubated for 1 h at room temperature. At the end of the incubation, the plates were washed 3 times with 300 μL of PBS/tween at 0.05 %, the cytokines controls and each one of the samples were added, and they were incubated for 2 h at ambient temperature. Each well was washed five times (0.05 % PBS/tween) and in order to observe the reaction, with detection antibody (1:250) and a conjugate of streptavidin-horseradish peroxidase (1:250) were added to each well; the plates were incubated for 1 h at room temperature. The wells were washed 7 times (0.05 % PBS/tween) and 100 μL of TMB substrate (Reagent Set BD Pharmingen, San Diego, CA, USA) was added and incubated for 30 min more (protected from the light). The reaction was stopped with 50 μL of 2 N H_2_SO_4_ solution and finally the quantification was performed in an ELISA reader at 450 nm (Dynatech, MR580). The concentration of different cytokines was determined using a standard curve in pg/mL. Macrophages that were stimulated with both polymyxin B sulphate (Sigma, St Louis CA; 10 μg/mL) and LPS 0111:B4 (Sigma Chemical Co., St. Louis, MO, USA; 100 ng/mL) were used as controls.

### NF-kB (pp65) translocation

The location and translocation of NF-kB protein in human macrophages adhered to glass slides, were analyzed using the Cellomics NF-kB kit (Thermoscientific) by an immunofluorescence assay following the manufacturer's instructions. The macrophages samples were treated separately with RPMI-1640 (10 % FBS), Pet (10 μg/mL) and 100 ng/mL LPS of *E. coli* 0111:B4 (Sigma Chemical Co., St. Louis, MO, USA). The preparations were incubated for 30 min at 37 °C in atmosphere of 5 % CO_2_. These were washed two times with PBS, permeabilized with Triton X-100 (0.1 %) and fixed with paraformaldehyde (4 %). The cells were blocked with 2 % BSA and normal horse serum (5 %) in Dulbecco-PBS solution for 30 min. Afterwards, they were incubated for another hour with NF-kB primary antibody (rabbit monoclonal anti-NF-kBpp65, Thermoscientific). The preparation was washed and incubated for another hour with staining antibody 1:2000 and secondary antibody 1:500 (Hoescht Dye and Dylight 488 Goat anti-rabbit). The fluorescence of macrophages to the different stimuli was observed using an epi-fluorescence Zeiss Axioskop 2 microscope, Zeiss Axiocam (Zeiss AG, Oberkochen, DE). The percentage of positive cells to pp65 was performed by counting 100 cells per field in four separated experiments.

### Statistical analysis

The results are reported as mean ± SD, of each experiment in a total of four donors in duplicate samples. Differences between the levels of cytokine were assessed using Wilcoxon test. Differences were considered significant if *P* was < 0.05.

## Results

### Pet purification

**Fig. 1:**
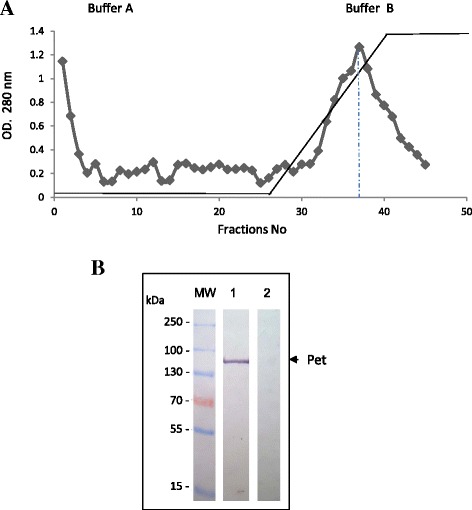
Purification of Pet protein by FPLC. Pet protein was purified from supernatant of the minimal clone HB101(pCEFN1).**a** Culture supernatant ammonium sulfateprecipitated was passed through a Q-Sepharose and FPLC columns. **b** The toxin specificity was analyzed by Western blot with anti-pet antibody (1:500) and the reaction visualized with rabbit anti-IgG antibody conjugated with alkaline phosphatase (1:5000). The reaction was evidenced adding BCIP/NBT. Lines: MW markers; 1 Pet toxin; 2 *Entamoeba histolytica* HMI-IMSS antigen

The Pet protein was obtained from the culture supernatant and identified between the 34 to 39 fractions of the cationic exchange column (Mono S HR 5/5 Pharmacia, USA) (Fig. 1a). The analysis of separated fractions (SDS-PAGE) showed a 104 kDa band with more than 90 % of purity. The Pet identity was confirmed by a Western-Blot test using specific antibodies (Fig. 1b).

### Pet effect on intestinal epithelium

**Fig. 2:**
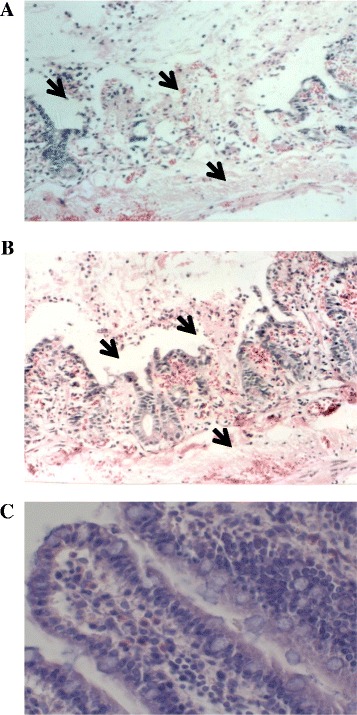
Histological Study of fractions obtained from rat ileal loops. Rat ileal loops inoculated with: **a** live O42EAEC bacteria (1.5 × 10^8^ UFC/mL); **b** 100 μg of purified Pet toxin and **c** PBS. The intestine preparations were Hematoxilin-Eosin stained and observed in light microscope (400X). Presence of hemorrhage (arrow), necrosis (arrow), ulceration and fibrinopurulent exudates (arrow) of the intestinal epithelium **a** and **b**. Non histological changes were observed in **c**

The histological analysis of tissue samples obtained from the intestinal ligated loops of rats, showed the presence of hemorrhage, necrosis, ulceration and fibrino purulent exudates in the intestinal epithelium samples in both, the inoculated with the live bacteria (Fig. 2a) and in those challenged with the purified Pet fraction (Fig. 2b). The preparations of the inoculated loop only with PBS did not showed alterations in the epithelium (Fig. 2c).

### Pet chemoattractant effect on macrophage

The different Pet concentrations (5 to 20 μg/mL) that were used in the chemotaxis assay activated the macrophage migration. The observed chemotactic effect was similar to the one which was induced by ZAS. It was also identified that the Pet effect depends on the concentration, the maximum migration was determinate to 20 μg/mL after 90 min of incubation (Table 1).

**Table 1:** Activation of human macrophages migration induced by Pet Protein in a Boyden chamber assay

Treatment	Cells number × fields^a^	Macrophages migration percentage
Hank’s solution BSA (0.2 %)	5 ± 2.5	16
ZAS	30 ± 3.4	100
Pet 5 μg/ml	10 ± 2.2	33
Pet 10 μg/ml	15 ± 5.1	50
Pet 20 μg/ml	22 ± 4.3	73

Values are mean ± SD

^a^Cells x fields refers the migrated cells counted in five randomly selected fields what were observed under a microscope set at 400 X magnification. The date of percentage presented are compared with the 30 cells observed with the stimulus treatment with ZAS (Positive control)

### Cytokines produced by macrophages

**Fig. 3:**
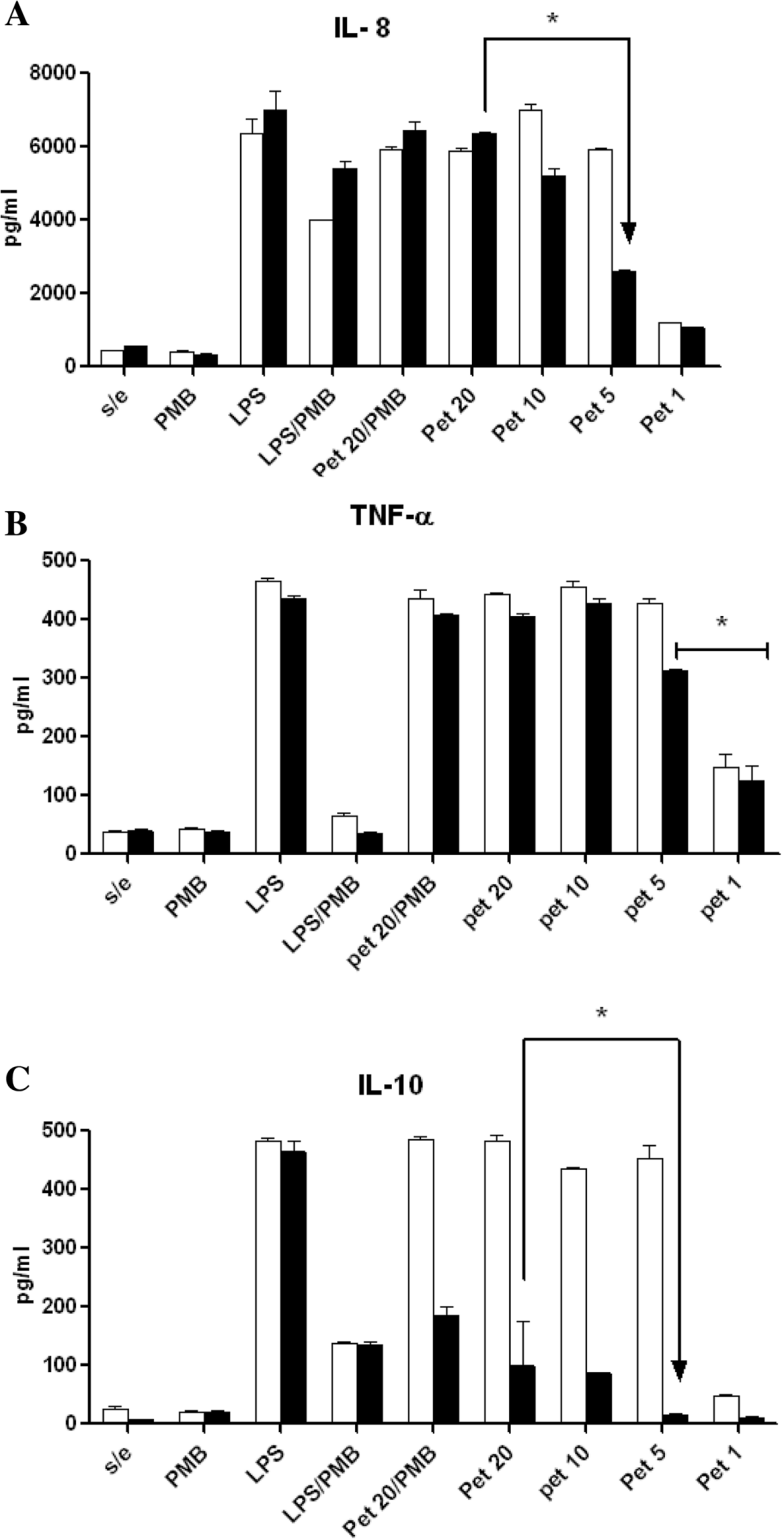
Cytokines production of MDM stimulated with Pet toxin from EAEC. Supernatants of MDM stimulated with Pet (1 to 20 μg/ml) were analyzed by ELISA test to determine the cytokines expression**. a** IL-8, **b** TNF-α and **c** IL-10. The MDM cells were treated with *E. coli*. 0111:B4 LPS (0.1 μg/ml); Polymyxin B (PMB), LPS and Polymyxin B (LPS/PMB), Pet (20 μg/ml) and Polymyxin B (Pet20/PMB), and untreated cells (s/e). The cytokine concentrations weredeterminedat 6(□) and 24 h (■) of stimulation. The results are the means ± of the standard deviation representative of four independent experiments. Significant *P* < 0.05 (*), as compared between 6 and 24 h of stimulation

The MDM (human peripheral blood monocytes-derived macrophages) treated with different Pet concentrations (5 to 20 μg/mL) induced the production of IL-8 from 6 and to 24 h of incubation. The maximum concentration of IL-8 was identified at 6 h in macrophages challenged with 10 μg/ml of toxin (Fig. 3a). The results showed one logarithmic increase when comparing the levels obtained in the cells without treatment, but with the similar values to the observed ones in the stimulated MDM with 0.1 μg/ml of LPS 0111:B4. The levels of IL-8 decreased at 24 h with the concentrations of 5 and 10 μg/ml, however, these cytokine remained high in the cells treated with 20 μg/ml of Pet (Fig. 3a). The effect of low concentrations of Pet (0.001 to 1 μg/ml) on the MDM was similar to the one obtained in the cells without treatment (data not shown).

In order to exclude that the activation of macrophages were due to an effect of residual LPS in Pet, both the toxin and the LPS 0111:B4 were treated with Polymyxin-B before the challenge. The results of the test showed a decrease of almost 41 and 25 % in IL-8 expression at 6 and 24 h in the MDM challenged with LPS treated with Polymyxin-B. The same test with Pet (20 μg/mL) treated with Polymyxin-B did not show changes in the IL-8 expression levels of the MDM treated with Pet alone (Fig. 3a). In this test, it was also identified that Pet activated the early expression of TNF-α (Fig. 3b) and the production of IL-10 with highest levels at 6 h, but with a decrease of more than 50 % after 24 h (Fig. 3c). On the other hand, analyzing the expression of IL-1β, IL-6 and IL-12 cytokines, it was found that Pet stimulates their expression using the concentrations from 5 to 20 μg/ml (data not shown).

### NF-kB (pp65) activation

**Fig. 4:**
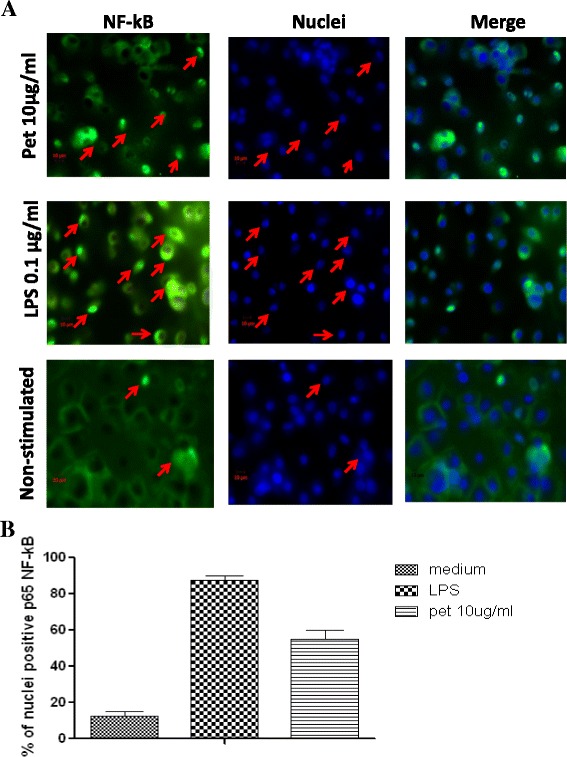
Immunofluorescence analysis of NF-kB activation. **a** Human macrophages were stimulated during 30 min with Pet (10 μg/ml) or LPS (Positive control) and non-stimulated. The nuclear translocation is determined (arrows) by the blue fluorescence (Hoechst staining) and the NF-kB p65 subunit (arrows) by its green fluorescence (Dylight 488). **b** Percentage of nucleic positive cells to p65. The results are representative of three independent experiments

The macrophages challenged with Pet also induced the activation and translocation of the NF-*k*B pp65 protein. This event was observed in more than 60 % of the cells after 30 min incubation. The same test in MDM stimulated with the LPS 0111:B4 showed activation in more than 90 % of cells. However, in non-treated MDM, the pp65 activation was observed in approximately 20 % of the cells (Fig. 4a, b).

## Discussion

The diarrhea caused by EAEC is the result of a complex interaction between the pathogen and the host. In this regard, it is interesting observed that in different clinical diseases induced by the enteroaggregative *E. coli* group, constantly is noted the activation of an inflammatory response [1, 2, 21–23], may be induced by LPS, fimbriae (AAF) or flagellin [8–10, 24]. On the other hand, the isolation of different *E.coli* strains that secreted SPATEs, have been observed in patients with IBD, cancer and Crohn’s disease [14]. In addition, in biopsies from patients with Crohn’s disease it has been identified inflammatory cells mainly macrophage subpopulations M1 [15, 16]. An interesting observation during a diarrhea outbreak associated with the EAEC infection, was the identification in the intestine autopsy of a child who passed away, the presence of inflammatory infiltration of lymphocytes and plasma cells, as well as the necrosis of the glandular epithelium and alterations associated with a clinical picture of enterocolitis (personal communication from Meyer-Gómez R. H. and Cravioto A.). In Pet toxin have been identified different biological properties [12, 13], some of them considered as the possibly cause of the intestinal alterations and the diarrhea caused by EAEC strains [11]. In this work, it was identified that the live Pet-producing bacteria as well as purified toxin fractions caused epithelium damage (Fig. 2a, b), alterations similar to those described in the intestine autopsy of the child previously referred.

Directed cell migration (chemotaxis) of neutrophils, monocytes and macrophages; i.e. inflammatory mediator cells, is an early step of the host innate immune response [25]. Some synthetic compounds, others that form a structural part of microorganisms (LPS, lipoteichoic acids, mycolic acids, glycolipids, lipoarabinomanana, polynucleotides, glycosylphosphatidylinositol, bacterial DNA, lipoproteins, flagellin), and cellular factors as IL-8, C5a of complement, formylated peptides (FMLP) and leukotrienes, possess adjuvant and activation effects of the innate immune system [24, 26–28]. Ulises et al. [29], mentioned that Pet is a chemoattractant factor of mononuclear cells (MN), the results of our study show that Pet activates chemotaxis of MDM, similar effect was obtained when the same cells are treated with ZAS, i.e. a compound of known chemotactic activity. The activation of cellular factors is induced as a protective mechanism against infection and colonization by microorganisms in epithelia [30]. If a secreted molecule by bacteria induces the migration of inflammatory cells, it could be expected as a suicide action of microorganism. However, the data obtained from studies using a mouse model infected with S. Typhimurium [31], shows that the intestinal inflammation induced by the bacteria infection alters the composition of the microbiota and suppresses its growth. So, it suggested that the inflammation is a positive factor for the survival and expansion of *Salmonella*.

An possible explanation as the bacteria can survive in unfavorable conditions, as is the case of an inflammatory process have been observed in *Serratia marcescens*, this bacteria secrete a protease of 56 kDa which has the property to inhibit the chemotactic function of C5a [32], similar observations were described in *E. coli* 0157:H7, this bacteria produce the metallo protease mucinase STcE type, which inhibits leukocyte and polymorphonuclear cells migration by mechanisms that involve participation of mucin-homologous glycoproteins [33].

EAEC besides Pet also secreted Pic protein, which also is member of the SPATEs family with mucinase activity [34] and capacity of decrease the chemotaxis and transmigration of leukocytes [35]. This feature in which two molecules expressed by the same bacteria with antagonistic effects, confirms the ability of some microorganisms to stay in the host using different properties that can be called of survival.

In the model of EAEC pathogenesis there are multiple factors which influence the inflammation scale [36, 37]. The Pet participation in the activation of the immune response only has been associated in chronic processes, but it has not been analyzed yet if the molecule is involved in the innate immune response. The results of our study propose Pet as a molecule which activates the production of pro-inflammatory cytokine IL-8 and TNF-α, both cytokines with important biologic effects since they participate in the regulation of the early inflammatory response and the activation of macrophages [38, 39].

Cytokine IL-10 with anti-inflammatory and immunosuppressive activity, as well as control of macrophage effector functions [40], was also detected in this work. The IL-10 production induced by Pet was identified in its highest levels at 6 h; although, this effect was not expected because it’s synthesis and kinetics is a delayed event. Also it was observed that the IL-10 values decreased almost 50 % after 24 h, this suggest that the early synthesis of IL-10 induced by Pet, could be related to a transient immunosuppression mechanism which allows to *E. coli* to survive in the intestine in the stage of adaptation to new niches. This transient effect has been described in other pathogens, in which it has been proposed that IL-10 mediated the negative regulation of the innate immune response, although this represents a risk for the host [41, 42]. The Toll-like receptors (TLRs) expressed in different cells including macrophages activate the extracellular signaling pathways after having recognized the PAMPs-type molecules; i.e. an event that leads to the release of NF-kB and their translocation to the nucleus. In the study, it was observed that Pet significantly induced the translocation of pp65 after 30 min of stimulation. Different reports showed that during the activation of the transcription factor NF-kB in the response to various stimuli by pathogens and bacterial molecules, its translocation from the cytoplasm to the nucleus is an event where the signaling pathway participates for the synthesis of several cytokines [42]. In their study, Benitez et al. [43], indicated the early expression of mRNA inflammatory mediators (IL-1, TNF-α, MIF, antagonist receptor of IL-1 [LTRa]) in the line of murine macrophages J774 activated by the Pet protein, through signaling pathways iKKαβ/NF-kB and MAP kinases. In epithelial cells derived from the intestines, has been reported the participation of the cytokeratin 8 (CK8) as the Pet receptor [44]. However, until now in the immune cells, the receptors activated by Pet are not clearly defined, in this study it was evaluated as one possibility the PAMP-type molecule through the TLR2 and TLR4 receptor using transfected HEk-293 cells (human embryonic kidney cells), however, the results showed that Pet is not a PAMP-type molecule (data not shown).

## Conclusion

In summary the results provide the fundamental findings in order to propose that in-vitro activation of the innate immune system induced by Pet involves the recruitment of human macrophages, the synthesis of pro-inflammatory cytokines and the NF-kB activation. These suggest the possible association of the Pet protein to polarization immune responses of M1 macrophages and also TH1 immune responses.

## Abbreviations

AMP, Ampicillin; BCIP/NBT, 5-bromo-4-cholo-3-indolyl-phosphate/nitro blue tetrazolium; CK8, Cytokeratin 8; CD40, Cluster of differentiation 40; EAEC, Enteroaggregative *Escherichia coli*; ELISA, Enzyme-Linked Immuno Sorbent Assay; EU, Endotoxin units; FITC, Fluorescein isothiocyanate; FBS, Fetal bovine serum; FMLP, formylated peptides; Hbp, Hemoglobin protease; ICAM-1, Intercellular Adhesion Molecule 1; IL, Interleukin; IKKαβ, IkB kinase αβ; IBD, Inflammatory bowel disease; LPS, Lipopolysaccharide; LB, Luria broth; LAL, Limulus amebocyte lysate; MAP, Mitogen-activated protein kinases; MIF, Macrophage migration inhibitory factor; MN, Mononuclear cells; MDM, Human peripheral blood monocytes-derived macrophages; NF-kβ, Nuclear factor kappa-light-chain-enhancer of activated B cells; PAMPs, Pathogen-associated molecular patterns; PBS, Phosphate buffer saline; Pet, Plasmid encoded toxin; Pic, Protein involved in colonization; PRRs, Pattern recognition receptors; SPATEs, Serine protease autotransporters of *Enterobacteriaceae*; SD, Standard deviation; SDS-PAGE, Sodium dodecyl sulfate polyacrylamide gel electrophoresis; STcE, Secreted protease of C1-esterase inhibitogr; TLRs, Toll-like receptors; TMB, Tetramethyl benzidine; TNF-α, Tumor necrosis factor alpha; Tsh, Temperature-sensitive hemagglutinin; ZAS, Opsonized zymosan

## References

[1] Estrada-Garcia T, Navarro-Garcia F (2012). Enteroaggregative *escherichiacoli*pathotype: a genetically heterogeneous emerging foodborne enteropathogen. FEMS Immunol Med Microbiol.

[2] Harrington SM, Dudley EG, Nataro JP (2006). Pathogenesis of enteroaggregative *Escherichia coli* infection. FEMS Microbiol Lett.

[3] Tzipori S, Montanaro J, Robins-Browne RM, Vial P, Gibson R, Levine MM (1992). Studies with enteroaggregative *Escherichia coli* in the gnotobiotic piglet gastroenteritis model. Infect Immun.

[4] Sainz T, Perez J, Fresan MC, Flores V, Jimenez L, Hernandez U, Herrera I, Eslava C. Histological alterations and immune response induced by Pet toxin during colonization with enteroaggregative *Escherichia coli* (EAEC) in a mouse model infection. J Microbiol. 2002;40:91–7.

[5] Steiner TS, Lima AA, Nataro JP, Guerrant RL (1998). Enteroaggregative *Escherichia coli* produce intestinal inflammation and growth impairment and cause interleukin-8 release from intestinal epithelial cells. J Infect Dis.

[6] Bouckenooghe AR, Dupont HL, Jiang ZD, Adachi J, Mathewson JJ, Verenkar P, Rodrigues S, Steffen R. Markers of enteric inflammation in enteroaggregative *Escherichia coli* diarrhea in travelers. Am J Trop Med Hyg. 2000;62:711–3. 10.4269/ajtmh.2000.62.711 11304060

[7] Jiang ZD, Greenberg D, Nataro JP, Steffen R, Dupont HL (2002). Rate of occurrence and pathogenic effect of enteroaggregative *Escherichia coli* virulence factors in international travelers. J Clin Microbiol.

[8] Steiner TS, Nataro JP, Poteet-Smith CE, Smith JA, Guerrant RL (2000). Enteroaggregative *Escherichia coli* expresses a novel flagellin that causes IL-8 release from intestinal epithelial cells. J Clin Invest.

[9] Harrington SM, Strauman MC, Abe CM, Nataro JP (2005). Aggregative adherence fimbriae contribute to the inflammatory response of epithelial cells infected with enteroaggregative *Escherichia coli*. Cell Microbiol.

[10] Strauman MC, Harper JM, Harrington SM, Boll EJ, Nataro JP (2010). Enteroaggregative *Escherichia coli* disrupts epithelial cell tight junctions. Infect Immun.

[11] Eslava C, Navarro-García F, Czeczulin JR, Henderson IR, Cravioto A, Nataro JP (1998). Pet, an autotransporter enterotoxin from enteroaggregative *Escherichia coli*. Infect Immun.

[12] Navarro-García F, Sears C, Eslava C, Cravioto A, Nataro JP (1999). Cytoskeletal effects induced by pet, the serine protease enterotoxin of enteroaggregative *Escherichia coli*. Infect Immun.

[13] Villaseca JM, Navarro-García F, Mendoza-Hernández G, Nataro JP, Cravioto A, Eslava C (2000). Pet toxin from enteroaggregative escherichia coli produces cellular damage associated with fodrin disruption. Infect Immun.

[14] Kotlowski R, Bernstein CN, Sepehri S, Krause DO (2007). High prevalence of *Escherichia coli* belonging to the B2 + D phylogenetic group in inflammatory bowel disease. Gut.

[15] Strober W, Fuss IJ (2011). Proinflammatory cytokines in the pathogenesis of inflammatory bowel diseases. Gastroenterology.

[16] Sepehri S, Khafipour E, Bernstein CN, Coombes BK, Pilar AV, Karmali M, Ziebell K, Krause DO. Characterization of *Escherichia coli* isolated from gut biopsies of newly diagnosed patients with inflammatory bowel disease. Inflamm Bowel Dis. 2011;17:1451–63. 10.1002/ibd.21509 21674703

[17] Taylor PR, Martinez-Pomares L, Stacey M, Lin HH, Brown GD, Gordon S (2005). Macrophage receptors and immune recognition. Annu Rev Immunol.

[18] Mosser DM, Edwards JP (2008). Exploring the full spectrum of macrophage activation. Nat Rev Immunol.

[19] Vial PA, Robins-Browne R, Lior H, Prado V, Kaper JB, Nataro JP, Maneval D, Elsayed A, Levine MM. Characterization of enteroadherent-aggregative *Escherichia coli*, a putative agent of diarrheal disease. J Infect Dis. 1988;158:70–9. 10.1093/infdis/158.1.70 2899125

[20] Bradford MM (1976). A rapid and sensitive method for the quantitation of microgram quantities of protein utilizing the principle of protein-dye binding. Anal Biochem.

[21] Huang DB, Mohanty A, Dupont HL, Okhuysen PC, Chiang T (2006). A review of an emerging enteric pathogen: enteroaggregative *Escherichia coli*. J Med Microbiol.

[22] Flores J, Okhuysen PC (2009). Enteroaggregative *Escherichia coli* infection. Curr Opin Gastroenterol.

[23] Hebbelstrup Jensen B, Olsen KE, Struve C, Krogfelt KA, Petersen AM (2014). Epidemiology and clinical manifestations of enteroaggregative *Escherichia coli*. Clin Microbiol Rev.

[24] Bendelac A, Medzhitov R (2002). Adjuvants of immunity: harnessing innate immunity to promote adaptive immunity. J Exp Med.

[25] Phillipson M, Kubes P (2011). The neutrophil in vascular inflammation. Nat Med.

[26] Kumar H, Kawai T, Akira S (2011). Pathogen recognition by the innate immune system. Int Rev Immunol.

[27] Porter SL, Wadhams GH, Armitage JP (2011). Signal processing in complex chemotaxis pathways. Nat Rev Microbiol.

[28] Wang JS, Lin HY, Cheng ML, Wong MK (2007). Chronic intermittent hypoxia modulates eosinophil- and neutrophil-platelet aggregation and inflammatory cytokine secretion caused by strenuous exercise in men. J Appl Physiol.

[29] Ulises HC, Tatiana G, Karlen G, Guillermo MH, Juan XC, Carlos E (2009). Peptide sequences identified by phage display are immunodominant functional motifs of Pet and Pic serine proteases secreted by *Escherichia coli* and *shigella flexneri*. Peptides.

[30] Frosali S, Pagliari D, Gambassi G, Landolfi R, Pandolfi F, Cianci R (2015). How the intricate interaction among toll-like receptors, microbiota, and intestinal immunity Can influence gastrointestinal pathology. J Immunol Res.

[31] Stecher B, Robbiani R, Walker AW, Westendorf AM, Barthel M, Kremer M, Chaffron S, Macpherson AJ, Buer J, Parkhill J, Dougan G, von Mering C, Hardt WD. *Salmonella enteric* serovar typhimurium exploits inflammation to compete with the intestinal microbiota. PLoS Biol. 2007;5:2177–89. 10.1371/journal.pbio.0050244 PMC1951780 17760501

[32] Oda T, Kojima Y, Akaike T, Ijiri S, Molla A, Maeda H (1990). Inactivation of chemotactic activity of C5a by the serratial 56-kDa protease. Infect Immun.

[33] Szabady RL, Lokuta MA, Walters KB, Huttenlocher A, Welch RA (2009). Modulation of neutrophil function by a secreted mucinase of *Escherichia coli* O157:H7. PLoS Pathog.

[34] Henderson IR, Czeczulin J, Eslava C, Noriega F, Nataro JP (1999). Characterization of Pic, a secreted protease of *shigella flexneri* and enteroaggregative *Escherichia coli*. Infect Immun.

[35] Ayala-Lujan JL, Vijayakumar V, Gong M, Smith R, Santiago AE, Ruiz-Perez F (2014). Broad spectrum activity of a lectin-like bacterial serine protease family on human leukocytes. PLoS One.

[36] Edwards LA, Bajaj-Elliott M, Klein NJ, Murch SH, Phillips AD (2011). Bacterial-epithelial contact is a key determinant of host innate immune responses to enteropathogenic and enteroaggregative *Escherichia coli*. PLoS One.

[37] Medeiros P, Bolick DT, Roche JK, Noronha F, Pinheiro C, Kolling GL, Lima A, Guerrant RL. The micronutrient zinc inhibits EAEC strain 042 adherence, biofilm formation, virulence gene expression, and epithelial cytokine responses benefiting the infected host. Virulence. 2013;4:624–33. 10.4161/viru.26120 PMC3906296 23958904

[38] Clark AR (2007). Anti-inflammatory functions of glucocorticoid-induced genes. Mol Cell Endocrinol.

[39] Parameswaran N, Patial S (2010). Tumor necrosis factor-α signaling in macrophages. Crit Rev Eukaryot Gene Expr.

[40] Sabat R, Grütz G, Warszawska K, Kirsch S, Witte E, Wolk K, Geginat J. Biology of interleukin-10. Cytokine Growth Factor Rev. 2010;21(5):331–44. 10.1016/j.cytogfr.2010.09.002 21115385

[41] Sing A, Rost D, Tvardovskaia N, Roggenkamp A, Wiedemann A, Kirschning CJ (2002). Yersinia V-antigen exploits toll-like receptor 2 and CD14 for interleukin 10-mediated immunosuppression. J Exp Med.

[42] Rahman MM, Mcfadden G (2011). Modulation of NF-kB signalling by microbial pathogens. Nat Rev Microbiol.

[43] Benítez A, Eslava C, Gonzalez C, Torres E (2011). Pet induces IL1, TNFα, MIF and IL1Ra through the IKKαβ/NFB pathway. Open Immun J.

[44] Nava-Acosta R, Navarro-Garcia F (2013). Cytokeratin 8 is an epithelial cell receptor for Pet, a cytotoxic serine protease autotransporter of enterobacteriaceae. M Bio.

